# Significantly Altered Serum Levels of NAD, AGE, RAGE, CRP, and Elastin as Potential Biomarkers of Psoriasis and Aging—A Case-Control Study

**DOI:** 10.3390/biomedicines10051133

**Published:** 2022-05-13

**Authors:** Adam Karas, Drahomira Holmannova, Pavel Borsky, Zdenek Fiala, Ctirad Andrys, Kvetoslava Hamakova, Tereza Svadlakova, Vladimir Palicka, Jan Krejsek, Vit Rehacek, Monika Esterkova, Helena Kovarikova, Lenka Borska

**Affiliations:** 1 Institute of Preventive Medicine, Faculty of Medicine in Hradec Kralove, Charles University, 500 03 Hradec Kralove, Czech Republic; karasad@lfhk.cuni.cz (A.K.); holmd9ar@lfhk.cuni.cz (D.H.); fiala@lfhk.cuni.cz (Z.F.); svadlakovat@lfhk.cuni.cz (T.S.); esterkovam@lfhk.cuni.cz (M.E.); borka@lfhk.cuni.cz (L.B.); 2 Institute of Clinical Immunology and Allergology, University Hospital, Faculty of Medicine in Hradec Kralove, Charles University, 500 03 Hradec Kralove, Czech Republic; andrys@fnhk.cz (C.A.); krejsekj@lfhk.cuni.cz (J.K.); 3 Clinic of Dermal and Venereal Diseases, University Hospital, 500 03 Hradec Kralove, Czech Republic; kveta@hamakova.cz; 4 Institute of Clinical Biochemistry and Diagnostics, University Hospital, Faculty of Medicine in Hradec Kralove, Charles University, 500 03 Hradec Kralove, Czech Republic; palicka@lfhk.cuni.cz (V.P.); kovarikovahe@lfhk.cuni.cz (H.K.); 5 Transfusion Center, University Hospital, 500 03 Hradec Kralove, Czech Republic; rehacekv@lfhk.cuni.cz

**Keywords:** psoriasis, inflammaging, biomarkers, RAGE, NAD, elastin, AGEs, CRP

## Abstract

Background: This study aims to investigate potential markers of psoriasis and aging, and to elucidate possible connections between these two processes. Methods: The serum samples of 60 psoriatic patients and 100 controls were analysed, and the levels of four selected parameters (AGEs, RAGE, NAD, and elastin) were determined using commercial ELISA kits. Serum C-reactive protein was assayed using an immune-nephelometry method. Findings: Among the patients, the levels of CRP, AGEs, and RAGE were all increased, while the levels of NAD were reduced when compared to the control group. A negative correlation between the levels of AGEs and NAD was found. A negative correlation between age and the NAD levels among the control group was observed, however among the patients the relationship was diminished. While there was no difference in the levels of native elastin between the patients and the controls, a positive correlation between the levels of native elastin and age and a negative correlation between the levels of native elastin and the severity of psoriasis were found. Conclusions: The results of our study support the notion of psoriasis and possibly other immune-mediated diseases accelerating the aging process through sustained systemic damage. The serum levels of CRP, NAD, AGEs, and RAGE appear to be promising potential biomarkers of psoriasis. The decrease in the serum levels of NAD is associated with (pro)inflammatory states. Our analysis indicates that the levels of native elastin might strongly reflect both the severity of psoriasis and the aging process.

## 1. Introduction

Psoriasis vulgaris is an incurable immune-mediated disease with skin symptoms [1]. Genetic predisposition and environmental factors determine the age of onset [2,3]. In the majority of patients, the affliction manifests in adulthood and the severity often worsens with progressing age [2,3,4].

The complex and partially unknown pathogenesis involves chronic systemic inflammation and oxidative stress [5,6]. Inflammation and oxidative stress are damaging interdependent processes–both can often induce each other [7]. Psoriasis is associated with lower life expectancy and age-related comorbidities, which include inflammatory arthritis, metabolic syndrome (including type 2 diabetes), and cardiovascular diseases [8,9,10,11,12,13].

Chronic low-grade inflammation is a hallmark of aging and plays a crucial role in the development of age-related disorders [12,13,14,15]. However, at the time of writing, more detailed understanding of the underlying molecular mechanisms is required [13]. Both the progression of psoriasis and the process of aging are associated with dysregulated immune cells and elevated levels of biomarkers of inflammation–such as C-reactive protein (CRP), tumor necrosis factor α (TNF-α), tumor necrosis factor β (TGF-β) and pro-inflammatory interleukins (including interleukin-6, IL-6)–enhanced expression and activity of matrix metalloproteinases, and neoangiogenesis [5,12,13,14,16,17,18,19]. Currently, there is a limited number of molecules eligible as clinical markers of psoriasis and aging [6,14,20]. We therefore aimed to investigate potential markers reflective of psoriasis and aging and to provide more detailed information on their possible relationships. Below, we describe our five chosen serum indicators of various relevant processes: CRP, advanced glycation endproducts (AGEs), receptors activated by AGEs (RAGE), elastin, and nicotinamide adenine dinucleotide (NAD).

CRP reflects systemic inflammation and is produced in response to the increased levels of IL-6 [14]. We primarily chose to investigate the levels of CRP for contextual evaluation of other parameters. Serum CRP is known from previous research to be higher in psoriatic patients, and is also associated with the severity of the disease [6]. This protein has been revealed to be associated with age of psoriatic patients and with advancing age in general [5,12,13,14,21,22].

AGEs are oxidant (highly reactive) molecules. Most AGEs are produced by both oxidation and non-enzymatic glycation. These glycoxidation products can be formed from various lipids, amino acids, and saccharides [23]. AGEs accumulation driven by oxidative stress and inflammation is a well-documented aspect of aging and is more rapid among psoriatic patients [24,25]. Receptors activated by AGEs (RAGE) are expressed on various cells including keratinocytes, endothelial, and immune cells. The bond between RAGE and its ligands activate immune cells, enhances metalloproteinases activity, increases the production of reactive oxygen species, and promotes the transcription of genes encoding proinflammatory cytokines. The inflammatory response then increases the production of AGEs, leading to even more inflammation in this pathological circle [25,26].

Elastin is an extracellular matrix protein necessary for elasticity and firmness of human tissues. It is an insoluble polymer of various isoforms of its soluble precursor tropoelastin, which matures into elastic fibres in extracellular space. Most elastin synthesis usually takes place during embryogenesis and in the neonatal period. Under physiological conditions, the turnover of insoluble elastin in adult life is very low and the remodelling of elastin fibres by elastases is reduced. The postnatal elastase activity can be stimulated by a proinflammatory environment and contribute to the pathogenesis of both immune-mediated diseases and age-related pathologies (including atherosclerosis) [27,28]. Cells associated with limited preserved elastogenesis in older age include skin fibroblasts, arterial smooth muscle cells, and endothelial cells [29].

The expression of elastin gene can be enhanced by various factors increased in both psoriasis and during aging, including TGF-β, TNF-α, and elastin fragments [30,31]. An increase in elastolysis with age and psoriasis is widely recognized, especially in skin and arteries [30,32,33].

NAD is a coenzyme and a metabolite directly and indirectly associated with a wide spectrum of essential cellular processes. These include DNA repair, regulation of epigenetics and gene expression, stem cell regeneration, regulation of oxidative stress, protein modification, and metabolic pathways such as glycolysis. The NAD level decline is an established feature of frailty, progeroid syndromes, and age-associated diseases (including atherosclerosis and diabetes) [15]. Changes in the NAD metabolism have been repeatedly associated with psoriasis [34,35], and NAD pharmacology is being considered as a direction of therapy for both age-related afflictions and psoriasis [35]. In psoriatic and frail patients, the potential benefits of administration of the precursor nicotinamide [36,37] or the vitamin B_3_ precursor [35] appear promising, as do the physiological ways of raising NAD levels [35].

## 2. Materials and Methods

### 2.1. Study Groups and Clinical Examinations

The group of 60 adult patients (PP) diagnosed with psoriasis (median; age 45.81 years) was compared to the control group (CG) of 100 adult healthy blood donors of similar age (median; age 48.7 years). Patients with inflammatory diseases, such as infectious diseases, malignancy, or inflammatory rheumatic diseases, those who were pregnant, and those using non-steroidal or anti-inflammatory medications were excluded from the study. The CG consisted of individuals with traits and habits comparable to those of the PP. The Psoriasis area severity index (PASI) was chosen for standardized evaluation of the clinical severity. Other clinical investigations included the determinations of age, smoking habit, clinical characteristics, weight, and body mass index (BMI).

### 2.2. Collection of the Blood Samples

The peripheral blood samples from all the participants mentioned above were collected from the cubital vein. The Vacutainer sampling tubes (Becton Dickinson, Franklin Lakes, NJ, USA) were used for collection. Whole blood samples were incubated for 30 min at room temperature. The samples were then centrifuged for 10 min at 1300× *g* (2500 rpm) and serum was isolated and stored under –70 °C.

### 2.3. Analysis of AGE

The levels of AGE in blood were determined using a commercial ELISA kit—Human Advanced Glycation End Products (AGEs) ELISA Kit, Cusabio (Houston, TX, USA). The product was used according to the manufacturer’s instructions. The limit of detection was 0.39–50  µg/mL.

### 2.4. Analysis of RAGE

The values of RAGE in serum were measured using ELISA kit—Quantikine ELISA Human RAGE Immunoassay (R&D Systems, Minneapolis, MN, USA). The product was used following the manufacturer’s instructions. The limit of detection was 78–5000 pg/mL.

### 2.5. Analysis of CRP

Serum C-reactive protein was assayed using an immune-nephelometry method. The analyser Immage 800 and diagnostics provided by the manufacturer Beckman Coulter (Brea, CA, USA) were chosen for the purpose of this study.

### 2.6. Analysis of Elastin

The concentrations of elastin in blood were analysed using commercial ELISA kit—Elastin SimpleStep ELISA Kit, ab239433, (Abcam; Cambridge, UK). The product was used according to the manufacturer’s instructions. The limit of detection was 0.19–12 ng/mL. The kit is designed for the in vitro quantitative measurement of the protein in its native form.

### 2.7. Analysis of NAD

The levels of NAD in serum were determined using Enzyme-linked Immunosorbent Assay Kit For Nicotinamide Adenine Dinucleotide (NAD) (Cloud-Clone Corp.; Katy, TX, USA). The product was utilized according to the manufacturer’s instructions. The limit of detection was 1235–100,000 ng/mL.

### 2.8. Statistical Analysis

To process the data, the Statistica software version 13.5.0.17 (TIBCO Software Inc.; Palo Alto, CA, USA) was chosen. For the evaluation of associations in regard to parameters, Spearman’s rank correlation test and Pearson’s correlation test were chosen. The intergroup differences were compared by using the T-test and the Mann-Whitney U test. The probability level (p) was acknowledged as significant when it was below the level of 0.05.

## 3. Results

### 3.1. Participant’s Data

A total of 160 individuals were enrolled in our study. The relationships between demographical findings, medians, and interquartile range in the PP and the CG are depicted in Table 1. The median age in the PP was 45.8 (N = 60, interquartile range 34.6–58.5). The median age in the CG was 48.7 (N = 100, interquartile range 39.0–56.1). The median weight in the PP was 80.0 (N = 60, interquartile range 67.0–90.0). The median weight in the CG was 83.5 (N = 100, interquartile range 68.5–95.0). The median BMI in the PP was 26.9 (interquartile range 23.7–30.7). The median BMI in the CG was 26.1 (interquartile range 23.7–29.7). As per the depiction in Table 1 and the description below it, there were no statistically significant differences among the examined traits.

The two groups were comparable in relation to most of the relevant traits and habits. While we wanted to exclude all heavy smokers, we had a higher number of individuals among the patients who had a limited smoking habit (55%). The analysis of significance did not show any confounding relationship between smoking and PASI (Spearman’s rank order correlation coefficient: r = 0.17, *p*-value: 0.178). The analysis of significance did not show any confounding relationship between smoking and CRP (Spearman’s rank order correlation coefficient: r = 0.084, *p*-value: 0.287126). The analysis (Spearman’s rank correlation test) proved the relationship between the serum levels of CRP and all other measured laboratory parameters to be insignificant among both the PP and the CG.

### 3.2. The Levels of Selected Parameters (CRP, NAD, AGE and RAGE, Elastin)

**Figure 1:**
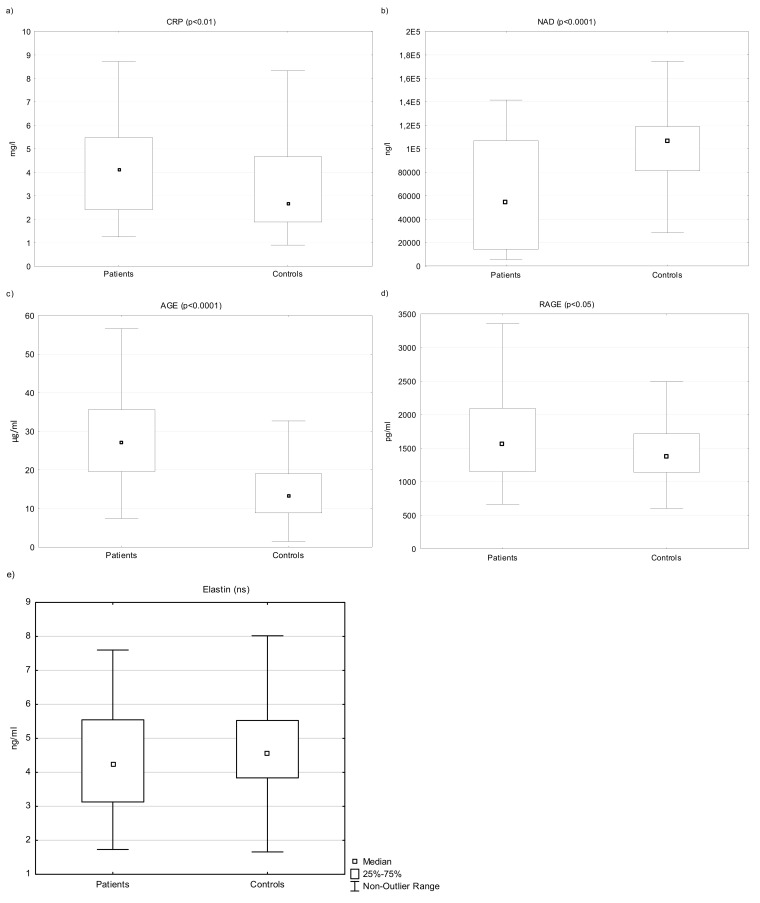
The levels of serum CRP (**a**), NAD (**b**), AGE (**c**), RAGE (**d**), and elastin (**e**) in patients and controls. Legend: An outlier is any data point value > 75th percentile + 1.5 × (75th percentile—25th percentile) or any data point < 25th percentile—1.5 × (75th percentile—25th percentile). ns = not significant.

The levels of CRP significantly differed between the groups. The levels were elevated in the PP (n = 60, median 4.12, interquartile range 2.41–5.50 mg/L) compared to the CG (n = 100, median 2.70, interquartile range 1.88–4.70; *p* < 0.01 (Figure 1a).

The levels of NAD were lower in the patients (n = 60, median 54,485 ng/L, interquartile range 14,368–106,775 ng/L) compared to healthy controls (n = 100, median 106,465, interquartile range 81,208–118,608 ng/L; *p* <0.0001) (Figure 1b).

The levels of both AGE and RAGE were significantly elevated in patients compared to the control group (n = 60, median AGE/RAGE 27.15 µg/mL/1564 pg/mL, interquartile range 19.60–35.6 µg/mL/1146–2096; n = 100, median AGE/RAGE 13.4 15 µg/mL/1375 pg/mL; interquartile range 8.85–19.0 µg/mL/1140–1718 pg/mL) *p* <0.0001/0.05) (Figure 1c,d).

The serum levels of elastin did not differ significantly between the patients and the controls overall (n = 60, median 4.24 ng/mL, interquartile range: 3.13–5.50 ng/L, CG: n = 100, median 4.60, interquartile range 3.84–5.50 ng/mL) (Figure 1e, Table 2).

However, the elastin levels, the age, and the severity of psoriasis (PASI) all significantly correlated with each other among the participants (Table 3).

### 3.3. Correlation among Measured Parameters

We also tested other possible relationships among the assessed laboratory parameters and measured clinical characteristics with correlation analysis (Spearman’s rank correlation test). Additionally, we evaluated whether the relationships are significantly different between the PP and the CG. The most relevant relationships and differences between the groups are depicted in Table 3.

The analysis of the relationship between age and CRP did not show a statistically significant difference between controls and patients (r = 0.141, *p* = 0.075); serum CRP and age correlated with each other only in the CG (r = 0.283, *p* = 0.028). Serum CRP and BMI positively correlated with each other in both groups (r = 0.413, *p* < 0.0001).

The analysis of the relationship between weight and CRP did show a statistically significant difference between controls and patients (r = 0.225, *p* = 0.004); serum CRP and weight significantly correlated with each other only in the PP (r = 0.402, *p* = 0.001).

Serum RAGE and BMI negatively correlated with each other in both groups (r = 0.280, *p* < 0.001).

Statistical significance was reached in regard to the negative correlation between the levels of AGE and NAD in both the CG and the PP (r = −0.050, *p* < 0.0001). The analysis of the relationship between age and AGEs did not show a statistically significant difference between controls and patients (r = 0.151, *p* = 0.056); serum AGEs and age significantly positively correlated with each other only in the CG (r = 0.283, *p* = 0.004).

Serum elastin and age positively correlated with each other in both groups (r = 0.053, *p* < 0.0001). The correlation between the levels of elastin and age was more significant in the PP (r = 0.799, *p* < 0.0001) than in the CG (r = 0.275, *p* < 0.005). The analysis also proved a significant negative correlation between the levels of elastin and PASI (r = −0.284, *p* = 0.027).

The analysis of the relationship between age and NAD did show a statistically significant difference between controls and patients (r = −0.238, *p* = 0.002); serum NAD and age significantly negatively correlated with each other only in the CG (r = −0.368, *p* < 0.001).

The analysis proved a significant positive correlation between PASI and age (r = −0.272, *p* = 0.035).

The analysis of the relationship between age and BMI did show a statistically significant difference between controls and patients (r = 0.259, *p* < 0.001); age and BMI significantly positively correlated with each other only in the PP (r = 0.397, *p* = 0.001) (Table 3).

As mentioned above, the most relevant relationships and differences between the groups are depicted in Table 3.

### 3.4. Sex Differences between Parameters

There was no difference in measured parameters between the sexes, except for elastin. Elastin values were significantly higher in women compared to men (men n = 89, median 4.3, interquartile range 3.18–5.20 ng/mL; women = 71, median 4.84, interquartile range 3.97–6.60; *p* < 0.01) (Table 4). There is no statistically significant difference between sexes in all the other studied parameters in general, nor in the subgroup of patients or in the controls.

## 4. Discussion

This study focused on psoriasis and aging and the possible link between these processes. The progression of psoriasis and the process of aging share many similarities associated with chronic inflammation and increased oxidative stress [6,10,38]. Significant differences between epigenetic and chronological age in psoriatic patients have been found by previous research [8]. Some physiological aspects of aging are enhanced and dysregulated among the psoriatic patients, including the accumulation of reactive compounds and the rate of apoptosis [5,25]. The expression of various molecules is known to reflect relevant forms of permanent systemic damage, but many such parameters may not prove to be significantly representative or practical in the clinical setting [6,14,20,39]. We aimed to investigate potential links in expressions of molecules associated with an immune-mediated disease and the aging process. When choosing the observable parameters described below (the levels of CRP, AGEs, RAGE, NAD, and elastin), we took into consideration the fact that according to the review written by Chung et al., current evidence emphasizes the mutual interactions between proinflammatory mediators [13]. In our study, we chose to collect blood specimens instead of skin samples because our primary aim was to contribute to the clarification of systemic pro-inflammatory state.

CRP is a well-established non-specific marker of acute systemic inflammation. As we expected, the elevations of CRP among the patients were observed, which corresponds with most of the previous research [10,22,40]. Our statistical analysis found no correlation between CRP and PASI, in accordance with some of the previous research [10,40]. This could either support the notion that CRP is not a reliable complementary test to PASI in regard to the evaluation of the severity of psoriasis, or that its utility in the clinical setting is limited only to the evaluation of the immediate level of systemic inflammation. We confirmed clear associations of CRP with BMI in both experimental groups. Most of the previous studies also reported positive correlations between CRP and BMI [41,42]. Additionally, our study has demonstrated a positive correlation between age and BMI among the patients. According to the review written by Chung et al., the importance of adipose tissue in regard to age-related systemic inflammation is well-established [13]. It is noteworthy that senescent preadipocytes secreting TNFα and IL-6 accumulate with age. The Spearman’s rank correlation test proved the relationship between the serum levels of CRP and all the other measured laboratory parameters to be insignificant among both the PP and the CG.

Although we found no difference between PP and CG elastin levels, we found a positive correlation between elastin and age in all participants, and a negative correlation between the levels of elastin and PASI in the PP. The previous research elucidating the elastolysis is well-established, but there is a limited amount of information regarding the elastogenesis [30,33].

Thus, our findings might suggest that aging may be associated with compensatory or pathological elastogenesis–various processes and factors accompanying aging and chronic inflammation can stimulate elastolysis and tropoelastin expression in skin and vascular system [30,31,43].

Increased remodelling or loss of elastin in skin due to ultraviolet radiation, hypoxia, chronological aging, or chronic inflammation could have been significant enough to stimulate elastogenesis in the aging and psoriatic participants [30,43,44].

The systemic pro-inflammatory state accompanying aging and psoriasis induces elastin remodelling in arteries, and elastin degradation products can stimulate elastogenesis and pathological neoangiogenesis [13,18,19,28,30,45,46,47]. Elastin could also be released into the bloodstream as a result of increased endothelial permeability, which is also associated with neoangiogenesis–or compromised structural integrity of the arterial wall or other blood vessels–which may be due to atherosclerosis. The incidence of atherosclerosis increases with age, and patients with psoriasis are also at higher risk of developing atherosclerosis, which is accompanied by increased and dysregulated elastogenesis leading to a disproportionate increase in elastin accumulation [27,33,48].

Interestingly, a negative correlation between PASI and elastin was revealed. Our results might imply that compensatory or pathological elastogenesis is less stimulated, dysregulated, or insufficient in psoriatic patients with more severe psoriasis, despite increased TGF-β1 expression, which promotes elastin expression. Previous research has demonstrated positive correlations between the levels of TGF-β1 and PASI, which we did not detect [49,50]. Furthermore, we found a statistically significant difference in elastin values between women and men. It is known that estrogen and progesterone modulate the structure of the extracellular matrix, its organization, turnover, and expression of proteins required for the synthesis of extracellular matrix compounds (collagen, elastin) [51]. A study by Zupan et al. described that 17β-estradiol treatment alone stimulated elastin synthesis, but only in women, not in men [52]. Thus, we suggest the differential expression of elastin between the sexes depends on sex hormones. Therefore, elastin is the only marker with levels that did not differ between controls and patients, and the only marker with levels that differed by sex.

Among the psoriatic patients, the levels of AGEs and RAGE were both increased. Association between the altered serum levels has also been observed in subjects with other inflammatory conditions [53,54]. Some of the previous research found a clear correlation between altered serum levels of AGEs and RAGE and the severity of the disease [55]. However, in our study, relationships between both AGEs and PASI and between RAGE and PASI were not statistically significant. The levels of AGEs have been investigated as a potential biomarker of aging by previous research,; in our study, we observed a correlation between age and AGEs levels only among the CG [56]. Palanissami et al. have published a review article confirming the notion of inflammation increasing both the generation of AGEs as well as the expression of RAGE [26]. Such upregulation has also been suggested in the context of psoriasis [57]. Interestingly, some of the previous research found the serum levels of RAGE to be lower in patients with psoriasis compared to the controls [55]. It is noteworthy that among some other immune-mediated diseases, a decrease in RAGE expression has been observed [58]. It must be remarked that confounding factors impacting the levels of RAGE include the possibility of unrecognized renal insufficiency and unreported medications [56].

As mentioned above, the serum NAD levels were significantly more decreased in the PP compared to the CG, which is consistent with the results of the studies in the review by Radenkovic et al. [35]. However, it is worthy of note that we found a significant relationship between age and the levels of NAD only among the CG. We presumed that the same correlation between age and NAD, only more pronounced, would also be found in psoriasis patients. No significant relationship between NAD and PASI or NAD and CRP was observed. This suggests that the NAD decrease is less gradual when enhanced by pathological chronic systemic inflammation, as opposed to a less intense pro-inflammatory state associated with physiological aging. Psoriasis might have diminished the relationship between the age and NAD levels existent in the CG. It is possible that psoriasis eventually stimulates some of the compensatory mechanisms described below. The lack of observed significant correlation between PASI and NAD might then be explained by PASI scoring primarily local (dermatological) symptoms.

NAD homeostasis is founded in a complex balance between its de novo synthesis, consumption, and generation from salvage pathways. NAD is constantly synthetized, altered, and recycled to maintain stable NAD levels in cells [15]. The decrease of NAD in patients and elderly controls might be associated with higher activation of NAD-consuming pathways. NAD consuming enzymes such as NAD^+^ glycohydrolases, CD38, sirtuins, and PARPs have clear links to accelerated aging. Both inflammation and aging lead to increased NAD consumption in cells. The consumption route enabled by PARPs can be increased due to oxidative damage to DNA associated with sustained low-grade inflammation [15].

The salvage of NAD from the precursor nicotinamide, nicotinamide mononucleotide, and nicotinamide riboside via the NAM salvage pathway appear the most likely to be the major contributor to the upregulation of NAD levels. The conversion of nicotinamide to nicotinamide mononucleotide is dependent on nicotinamide phosphoribosyltransferase (NAMPT). Inflammation is known to impede this recycling mechanism, and activators of NAMPT are considered potential clinical targets of age-related diseases [15,59].

As demonstrated above, numerous established mechanisms of NAD decrease are relevant in the context of our study. It is possible that a longer duration of psoriasis and its influence on NAD metabolism might provide more time for compensatory mechanisms to be stimulated, therefore a positive correlation between age and the NAD levels among the patients would not be observed.

The possible influence of increased glycolysis might directly causally contribute to the correlation between NAD and AGEs observed among both the PP and the CG in the sense of increased utilization. However, the indirect influence of AGEs through oxidative stress might prove to be a more significant contributing factor [60,61].

## 5. Conclusions

The results of our study support the notion of psoriasis and possibly other immune-mediated diseases accelerating the aging process through sustained systemic damage. The chronic inflammation, dysregulation of metabolism, and increased oxidation appear to play significant interconnected roles. Our results confirm known and suggest new markers of psoriasis and aging. The studied parameters could prove to be particularly valuable in regard to risk assessment of age-related disorders among psoriatic patients in future studies. Our data demonstrate that the decrease of the NAD levels is more significant in psoriatic patients than in aging controls, and suggest that compensatory mechanisms develop in psoriatic patients over time. The discovery of the relationships between the levels of native elastin, aging, and the severity of psoriasis is a novelty with promising potential for clinical practice, but further research is needed to confirm the observed relationships in larger groups of geriatric and psoriatic patients.

## Figures and Tables

**Table 1:** Demographic findings expressed as medians with interquartile ranges (Q1,Q3).

Variable	Patients (PP)				Controls (CG)				
	N	Median	Q1	Q3	N	Median	Q1	Q3	*p*-Value
Age	60	45.8	34.6	58.5	100	48.7	39.1	56.1	NS
Weight	60	80.0	67.0	90.0	100	83.5	68.5	95.0	NS
BMI	60	26.9	23.7	30.7	100	26.1	23.7	29.7	NS

NS = non-significant.

**Table 2:** Laboratory findings expressed as medians and interquartile ranges (Q1,Q3).

Variable	Valid N	Median	Q1	Q3	*p*-Value
Elastin					
Patients	60	4.24	3.13	5.50	NS
Controls	100	4.60	3.84	5.50	
NAD					
Patients	60	54,485	14,368	106,776	<0.0001
Controls	100	106,465	81,208	118,608	
CRP					
Patients	60	4.12	2.41	5.50	<0.01
Controls	100	2.70	1.88	4.70	
AGEs					
Patients	60	27.15	19.60	35.60	<0.0001
Controls	100	13.40	8.85	19.00	
RAGE					
Patients	60	1564	1146	2096	<0.05
Controls	100	1375	1140	1718	

NS = non-significant.

**Table 3:** Relationships among the tested parameters and differences between the PP and the CG.

Pair of Variables	Patients (PP)			Controls (CG)			All Groups		
	Valid	Spearman	*p*-Value	Valid	Spearman	*p*-Value	Valid	Spearman	*p*-Value
Age & PASI	60	−0.27214	<0.05						
Age & BMI	60	0.39711	<0.01	100	0.130206	NS	160	0.259092	<0.001
Age & AGE	60	−0.0252	NS	100	0.283483	<0.01	160	0.151202	NS
Age & Elastin	60	0.7995	<0.0001	100	0.275487	<0.01	160	0.53016	<0.0001
Age & NAD	60	−0.11512	NS	100	−0.36829	<0.05	160	−0.23827	<0.05
Age & CRP	60	0.283301	<0.05	100	0.061266	NS	160	0.141147	NS
PASI & Elastin	60	−0.28479	<0.05						
AGE & NAD	60	−0.4014	<0.05	100	−0.3729	<0.05	160	−0.50575	<0.05

NS = non-significant.

**Table 4:** Sex differences expressed as medians and interquartile ranges of (Q1,Q3).

Variable	Valid N	Median	Q1	Q3	*p*-Value
Elastin					
Men	89	4.30	3.18	5.20	*p* < 0.01
Women	71	4.84	3.97	6.60	
NAD					
Men	89	105,270	48,587	115,360	NS
Women	71	98,250	52,730	43,477	
CRP					
Men	89	3.00	2.04	4.70	NS
Women	71	2.94	2.05	5.30	
AGEs					
Men	89	16.50	10.90	25.90	NS
Women	71	18.20	10.30	29.20	
RAGE					
Men	89	1368	1126	1769	NS
Women	71	1485	1185	2032	

NS = non-significant.

## Data Availability

The data supporting published results are available from the corresponding author if requested.
